# The Effectiveness of Computerized Cognitive Training Combined With Whole Body Cryotherapy in Improving Cognitive Functions in Older Adults. A Case Control Study

**DOI:** 10.3389/fpsyt.2021.649066

**Published:** 2021-06-25

**Authors:** Adrianna Senczyszyn, Renata Wallner, Dorota Maria Szczesniak, Mateusz Łuc, Joanna Rymaszewska

**Affiliations:** Department of Psychiatry, Wroclaw Medical University, Wroclaw, Poland

**Keywords:** mild cognitive impairment, cognitive functions, whole body cryotherapy, computerized cognitive training, dementia, aging, non-pharmacological methods

## Abstract

**Objectives:** Subjective Cognitive Decline (SCD) and Mild Cognitive Impairment (MCI) are common in elderly population, and constitute a high-risk group for progression to dementia. Innovative, complex, and engaging non-pharmacological methods of cognitive stimulation, implementable at this stage, are needed. The aim of the study was to determine the effect of Computerized Cognitive Training (CCT) combined with Whole Body Stimulation (WBC) on cognitive functions of older adults with SCD and MCI.

**Methods:** A 9-week single-blind pre/post case control trial was conducted. The study enrolled 84 adults aged 60 or older, allocated to one of two intervention groups: EG; CCT with psychoeducation, EG2; CCT with psychoeducation and 10 WBC sessions, or the control group (CG), which comprised patients receiving usual care. The primary outcome measures were cognitive functions evaluated with MoCA scale and several other neuropsychological tools. Depressive symptoms assessed with the GDS scale constituted the secondary outcome measures.

**Results:** The results show evidence for increased performance in the assessment of general cognitive functioning in both EGs (*p* ≤ 0.05). Significant improvement was also visible in several cognitive domains, such as verbal fluency (EG1 & EG2), learning ability and immediate memory (EG1 & EG2), delayed memory (EG2), attentional control (EG1), and information processing (EG2) (*p* ≤ 0.05). However, only in the group with combined interventions (CCT + WBC) the participants presented significantly less depressive symptoms (*p* ≤ 0.05).

**Conclusions:** The results of the study suggest that CCT, especially in combination with WBC, might be a practical and effective method of improving cognitive performance. Moreover, this combination leads to a reduction of depressive symptoms.

## Introduction

The world's population is aging rapidly. According to World Population Prospects: the 2019 Revision, by 2050, one in four persons living in Europe and Northern America could be aged 65 or above, and the number of persons aged 80 is expected to triple (1). Social and health systems in both developing and developed countries are not prepared for such demographic change. The increase in life expectancy results in higher incidence and prevalence of age-related cognitive decline, which involves neurodegenerative changes, such as loss of neurons and a decrease in the production of neurotransmitters. These changes are manifested by general slowness of psychomotor skills (2) along with reduced memory, attention, executive functions, and reasoning capacities (3). So far, there are no curative pharmaceutical methods of treatment for cognitive impairment and dementia (4). Alarmingly, due to the sense of diminished mental capabilities, older adults often exhibit withdrawal from many areas of activity (e.g., professional, social, educational) and a decrease in their independence and self-esteem (2, 5) as well as the stigma phenomenon (6). For these reasons, they need to be provided with opportunities to avoid social isolation and loss of interest (7).

Therapeutic methods of cognitive and social stimulation are being sought in order to achieve these goals. In our study, we investigated whether Computerized Cognitive Training (CCT) combined with Whole Body Cryotherapy (WBC) could constitute effective methods of cognitive stimulation.

### Computerized Cognitive Trainings

Computerized Cognitive Trainings (CCTs) constitute a relatively new non-pharmacological approach to cognitive stimulation. Studies on their effectiveness suggest that participation in cognitively and socially engaging activities can slow down the deteriorative processes (8–10). CCTs are conducted as group or individual training sessions with the use of electronic devices (e.g., computer, laptop, tablet, etc.,), and consist of various assignments intended to stimulate selected cognitive functions, such as attention, verbal and non-verbal memory, working memory, processing speed, or visuospatial skills. Moreover, CCTs include novel learning experiences–participants who are not familiar with digital devices need to acquire new skills, which in turn may lead to the creation of new neural pathways (11, 12). CCTs are characterized by a high level of control over the session, the use of standardized tasks and involvement of participants in planned, customizable, and structured activities (13).

There are two basic assumptions underlying CCTs. Firstly, training a particular cognitive function regularly can help maintain or increase the level of its efficiency. Secondly, the effects of the training can be generalized and lead to an improvement in the general functioning of patients (transfer of the trained skills) (14). CCTs are often accompanied by psychoeducational activities, e.g., (15, 16) during which the characteristics of cognitive domains as well as specific learning techniques that are beneficial for them are being discussed.

### Whole-Body Cryotherapy

Another promising method of non-pharmacological treatment of cognitive decline is called Whole-Body Cryotherapy or Cryostimulation (WBC). WBC involves a repetitive, short-term (up to 3 min) exposure to extremely low temperatures, and is nowadays widely used to relieve symptoms of various ailments in the course of which inflammation, muscle spasms, chronic pain, and swelling are observed (17–20). Systematic review revealed that WBC may exert beneficial effects on the lipid profile in terms of lowering the levels of total cholesterol, LDL, and triglycerides (21). Preliminary studies suggest that it may also be an effective method of improving cognitive functioning, especially memory processes (22). According to current knowledge, vascular malfunction, mitochondrial damage, oxidative stress and inflammatory response contribute to the development of cognitive deterioration, and WBC might be a response to these processes (20, 23–26). Recent studies suggest anti-inflammatory, anti-analgesic, metabolic, hormonal, and anti-oxidant effects of this therapy based on the underlying physiological responses (21, 27, 28). Since WBC addresses some of the aforementioned processes contributing to cognitive deterioration, we postulate that it might be considered as a relatively safe and low-cost method of its prevention. However, the exact mechanisms underlying the influence of cryogenic temperature intervention on cognitive functions requires further investigation (21, 29).

## Materials and Methods

### Study Design

**Figure 1:**
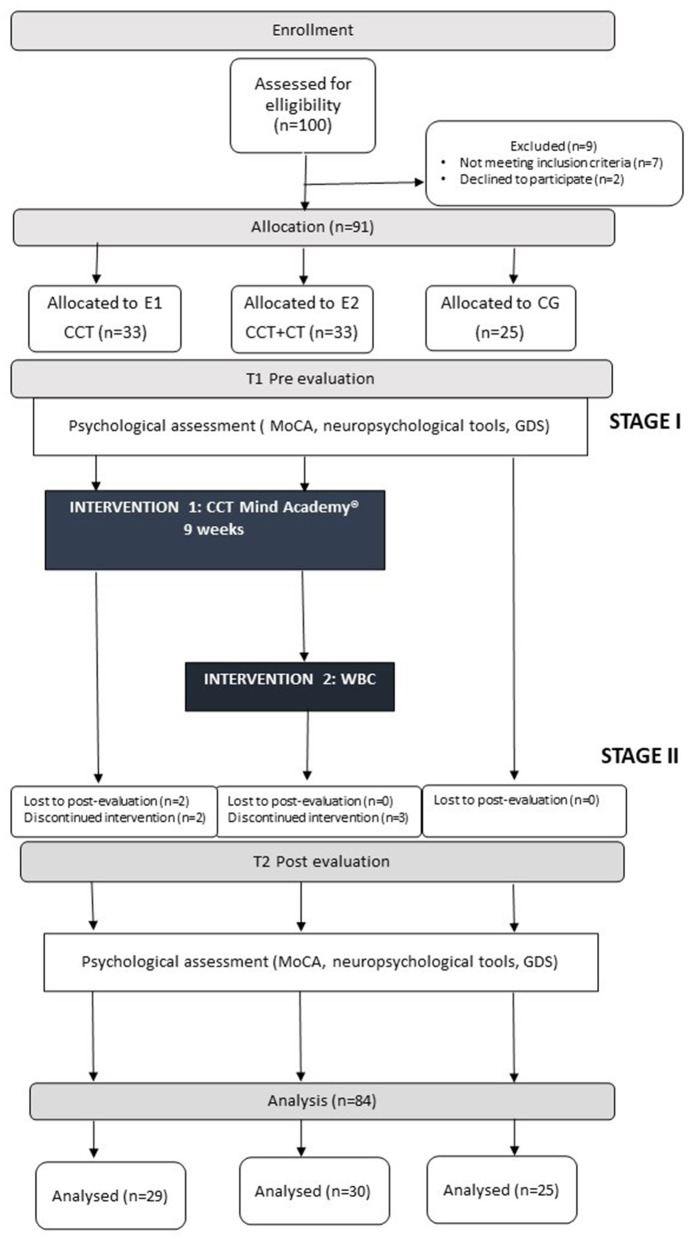
The consort flow diagram modified for non-randomized trial design.

The study was designed as a pre/post case control trial. There were two intervention groups and one control group: EG1–Experimental Group with Computerized Cognitive Training and EG2– Experimental Group with Computerized Cognitive Training and Whole-Body Cryotherapy, and CG– Control Group–which were comprised of community dwelling older people. The groups were matched for age, gender, and level of cognitive functioning. Cognitive assessment in EGs was conducted twice: in the first week before the first session of CCT (T1) and in the week after the last CCT (T2). CG was also assessed twice, with a 9-week break period. Participants who were present at least 80% of the intervention sessions were deemed adherent. The research protocol for this trial was reviewed and approved by the Bioethical Committee (number KB61/218) at Wroclaw Medical University. The consort flow diagram modified for non-randomized trial design is presented in Figure 1.

### Subjects

One hundred community-dwelling elderly people from the Lower Silesian Public Library in Wroclaw were enrolled in the study. All of them had subjective cognitive impairments, which were either confirmed in objective neuropsychological assessment (participants with MCI) or not (participants with SCD). Theinclusion criteria for CCT were: age ≥ 60 years, basic computer knowledge, cognitive norm/mild cognitive impairment (MCI). Cognitive functioning was assessed with the Montreal Cognitive Assessment, MoCA, and the cut-off points were: >26 for norm, 26–20 for MCI, ≤ 19 for dementia (30, 31). The exclusion criteria included more advanced cognitive decline (19 ≤ MoCA), inability to understand questions and written information, psychosis, standard contradictions to use WBC (e.g., acute respiratory diseases, acute cardiovascular disease like coronary disease, circulatory insufficiency, unstable hypertension, cold intolerance, claustrophobia, cryoglobulinemia, cancer, deep vein diseases, hypothyroidism, neuropathies, purulent skin differences, Reynaud disease), and previous exposition to WBC treatment.

Seven people did not meet the inclusion criteria (≤19 MoCA), two refused to participate. Those among the 91 people who were unable to attend regular CCT were assigned to the control group (*n* = 25). As a result, 66 participants were cognitively able and willing to commit to CCT/CCT + WBC. The participants from this group in whom there were no standard contraindications to the use of WBC were assigned to CCT + WBC. As a result, the EGs were equinumerous (EG1 *n* = 33, EG2 *n* = 33). Over the course of the intervention, seven participants dropped out (four due to some minor health problem, three went on vacation), leaving final sample size of 84. They received detailed oral and written information regarding the design of the study, the possibility to resign at any of its stages and information on the anonymity of the study. Before the study began, the participants provided their written consent to its terms. Additionally, those who underwent WBC were informed about the procedure in the presence of a clinician. EGs and CG were homogeneous in terms of sociodemographic variables, MoCA, and Geriatric Depression Scale (GDS) (32, 33).

### Intervention

CCT was conducted in a group format comprising 8–12 participants per group. Participants from EGs attended the Lower Silesian Public Library once a week for 1.5-h sessions, for 9 weeks. Each session comprised of 3 components:

(1) psychoeducation: promotion of knowledge about healthy brain aging, brain training possibilities, learning and compensation strategies, etc.,;
(2) Computerized Cognitive Training: individual computer tasks targeting specific cognitive functions;
(3) interactive group tasks: including tasks introduced to increase the stimulation of cognitive functions and social interactions between the participants.

What is more, each session was followed by a homework assignment corresponding to the main subject of a particular group task. The CCT program referred to the concept of cognitive stimulation (14, 34), which is an approach based on engaging participants not only in a range of cognitive tasks, but also exercises conducted in groups, introduced to promote social activation. The cognitive tasks used in the project were selected from the *Mind Academy*® cognitive training (Formsoft®, Wroclaw, Poland), an educational program created to improve mental capacity in the elderly. The cognitive exercises offered by *Mind Academy*® target a whole spectrum of cognitive domains, such as attention, immediate and delayed memory, mental flexibility, processing speed, categorization, reaction time, and visuospatial skills. During each training session, the participants followed an individualized training course (the difficulty level of the tasks was adapting to the progress made by each person). Each session was conducted and supervised by a certified trainer who conducted CCTs in accordance with the instructions of a standardized 9-week training schedule. The second type of procognitive stimulation was WBC. The cryotherapy chamber (CR 2002, Wroclaw type), cooled by liquid nitrogen, had two rooms: the vestibule/antechamber (with the temperature of −60°C, and the proper chamber with temperature from −110°C on the first day to −130°C on the following days).

Cryotherapy sessions lasted 2 min in the main chamber with 30 s extra for adaptation in the vestibule before and after the proper session (35). The chamber was used by 5–6 people at the same time. The participants wore minimal, woolen or cotton clothing. To reduce the risk of injuries caused by the cold and to protect their limbs and heads, the participants put on gloves, high-knee socks, dry shoes, beanies, and mouth masks. Before every WBC session, each participant was examined by a physician who also measured their blood pressure.

### Instruments

The primary outcome measures were general cognitive functions and selected domains of cognitive functioning listed below. Depressive symptoms constituted the secondary outcome. Each cognitive domain (attention and working memory, psychomotor speed, visuospatial/constructional functions, executive functions, language skills, verbal and visual memory) was evaluated by an independent psychometric tool. To reduce the risk of test performance improvement after CCT, as a result of learning the effect of repeated assessments, parallel versions of MoCA and RBANS, were used.

Psychometric tools used to assess participants' cognitive performance:

#### Primary Outcomes

##### General Cognitive Performance

- The Montreal Cognitive Assessment Scale (MoCA) (score range: 0–30). Cut-off points (>26 for norm, 26–20 for MCI, ≤ 19 for dementia) were taken from the first Polish language version (31).

##### Attention and Working Memory

- Digit Span from MoCA (score range: 0–2) assessing attentional control, sustained attention and immediate memory.
- Serial Sevens from MoCA (score range: 0–2) measuring working memory.

##### Psychomotor Speed

- The Trail Making Test A (TMT: part A) (36, 37) (time in s′) measuring psychomotor speed.

##### Executive Functions

- The Stroop Color and Word Test (SCWT) (38) (time in s′) for the assessment of the ability to inhibit cognitive interference.
- Phonemic Fluency Test (36) letter “k” (T1), “m” (T2), (number of words in s′) measuring phonemic verbal productivity and executive control.

##### Language Skills

- Semantic Fluency “fruits and vegetables” (T1), “pieces of clothing” (T2) from the Repeatable Battery for the Assessment of Neuropsychological Status (RBANS) (39) (score range: 0–40) for the assessment of semantic verbal productivity.
- Picture Naming from RBANS (score range: 0–10) assessing mental lexicon searching and naming skills.

##### Verbal Memory

- List Learning from RBANS (score range: 0–40) measuring the learning ability and immediate episodic memory.
- List Recall from RBANS (score range: 0–10) for the assessment of delayed episodic memory.
- Logical Memory I from RBANS (score range: 0–24) measuring the learning ability and immediate logical episodic memory.
- Logical Memory II from RBANS (score range: 0–12) for the assessment of episodic logical delayed memory.

##### Visual Memory

- Figure Recall from RBANS (score range: 0–20) measuring delayed visual memory.

##### Visuospatial/Constructional Function

- Figure Copy from RBANS (score range: 0–20) assessing planning and organization.
- Line Orientation from RBANS (score range: 0–20) measuring visual-spatial orientation.

#### Secondary Outcome

##### Depressive Symptoms

- The Geriatric Depression Scale, 30-item version, (score range: 0–30) for the assessment of depressive symptoms. The cut-off points were as follows: norm: 0–9, mild depressives: 10–19, severe depressives: 20–30.

### Statistical Analysis

The D'Agostino-Pearson test and visual assessment were used to analyse the normality of the data. Demographic characteristics at baseline were compared using the Fisher exact test for independent samples (gender, place of residence, education, work, marital status) and the Kruskal-Wallis tests (age). Primary and secondary outcome measures obtained in baseline and follow-up were compared using the Fisher exact test for independent samples (qualitative variable) and Kruskal Wallis (quantitative variables). Multiple factor analysis of differences between groups and changes in time in test results was obtained using linear mixed models in the case of quantitative characteristics surveys, or cumulative link model in the case of ordinal characteristics. The level of statistical significance was set at 0.05. Calculations were made using the R for Windows package (version 3.6.1).

## Results

### Demographic Characteristics

Eighty-four people with MCI or SCD completed the study–59 in experimental groups (*n* = 29 in EG1, *n* = 30 in EG2) and 25 in the control group. The mean age of EG1 participants was 77.1 (±5.7), EG2 = 71.1 (±6.9), and CG = 69.4 (±6.3). Married patients constituted 60% of EG1, 51.72% of EG2, and 40% of CG. Most patients were female– 89.7% in EG1, 76.6% in EG2, and 72% in CG. At least half of all participants had higher education: EG1 = 55.1%, EG2 = 56.7%, CG = 64%. The majority of people enrolled in the study lived in big cities (100% in EG1, 93.3% in EG2, and 96% in CG) and were retired (96.5% in EG1, 100% in EG2, and 100% in CG). EGs and CG were similar in terms of global cognitive status [MoCA mean scores: EG1 = 25 (±2.3 SD), EG2 = 25.1 (±2.7 SD), CG = 25.4 (±2.7 SD)] and depressive symptoms [GDS mean scores: EG1 = 8.8 (±5.7 SD), EG2 = 7.7 (±5.1 SD), CG = 8.4 (±5.7 SD)] measured before the interventions. Detailed demographic characteristics are presented in Table 1.

**Table 1:** Baseline characteristics of the participants.

**Characteristics**	**EG1 (CCT)**	**EG2 (CCT + WBC)**	**CG**	***p*-value**
	**(*n* = 29)**	**(*n* = 30)**	**(*n* = 25)**	**(*p* < 0.05)**
Age (mean, SD)	71.7 (5.7)	71.1 (6.9)	69.4 (6.3)	0.27
Women (%)	26 (89.7%)	23 (76.6%)	18 (72%)	0.25
University education (%)	16 (55.1%)	17 (56.7%)	16 (64%)	0.29
Married (%)	18 (60%)	15 (51.72%)	10 (40%)	0.35
Town dweller (%)	29 (100%)	28 (93.3%)	24 (96%)	0.16
Retirement (%)	28 (96.5%)	30 (100%)	25 (100 %)	0.64
GDS (mean, SD)	8.8 (5.5)	7.7 (5.1)	8.4 (5.7)	0.83
MoCA (mean, SD)	25 (2.3)	25.1 (2.7)	25.4 (2.7)	0.89

*Values are given as means (SD) or n (%). EG1 (CCT), experimental group with Computerized Cognitive Training; EG2 (CCT + WBC), experimental group with Computerized Cognitive Training and Whole Body Cryotherapy; CG, control group; MoCA, Montreal Assessment Cognitive Scale; GDS, Geriatric Depression Scale*.

### Cognitive Outcomes

#### Primary Outcomes

##### Global Cognitive Functioning

The multiple factor analysis showed significant time by group interaction in favor of EGs: EG1 (CTT) and EG2 (CCT + WBC) in comparison to CG for general cognitive functioning (MoCA: EG1 *p* = 0.002, t = 3.137, EG2 = *p* = 0.000, t = 4.023). However, no significant difference between EG1 and EG2 was observed (*p* = 0.373, t = −0.895). Moreover, taking into account the clinical interpretation of the scores obtained by subsequent participants, it is observed that over time, in both experimental groups, some patients (EG1 = 4 people, EG2 = 12 people) changed the category from MCI (MoCA: 20–26) to cognitive norm (MoCA > 26), wherein there was no such change in categorical adherence in CG. The results of selective cognitive function subtests were more ambiguous.

##### Attention and Working Memory

Primarily, in the time by group attention and working memory assessment, the participants in EG1 improved their performance in the Digit Span subtest from MoCA (*p* = 0.041, OR = 8.041), but the participants in EG2 did not (*p* = 0.232, OR = 3.227). However, the overtime comparison between the experimental groups in Digit Span was insignificant (*p* = 0.350, OR = 0.401). In the latter subtest–Serial Sevens from MoCA–the time by group difference was insignificant in both EGs (EG1 *p* = 0.773, OR = 1.253, EG2 *p* = 0.993, OR = 0.994).

##### Psychomotor Speed

In the assessment of psychomotor speed conducted with the use of the Trail Making Test: part A only the difference between EG2 and CG measured over time was statistically significant (EG2 *p* = 0.035, t = −2.148), while in EG1 the difference was close to significance (EG1 *p* = 0.083, t = −1.753). The difference between EGs was insignificant (*p* = 0.778, t = −0.270).

##### Executive Functions

With regard to executive functions, the improvement in the group by time assessment was statistically significant (EG1 *p* = 0.000, t = 3.640, *p* = 0.003, t = 3.042) only in Phonemic Fluency from MoCA. Nevertheless, the group by time over time comparison between EGs was insignificant (*p* = 0.517, t = 0.651).

##### Language Skills

Similarly, only in one of the tools used for the language skills group by time assessment the difference was statistically significant (Semantic Fluency from RBANS EG1 *p* = 0.010, t = 3.640, EG2 *p* = 0.010, t = 2.619). However, the same effect did not persist in the EGs over time comparison (*p* = 0.953, t = 0.059).

##### Verbal Memory

In the case of verbal memory, two out of four subtests supported the hypothesized group-time interaction in at least one EG: Logical Memory I from RBANS (EG1 *p* = 0.007, t = 2.770, EG2 *p* = 0.004, t = 2.992) and List Recall from RBANS (EG1 *p* = 0.751, *p* = 0.319, EG2 *p* = 0.025, t = 2.280). In the List Recall subtest the over time difference between EG1 and EG2 was statistically significant in favor of EG2 (*p* = 0.008, t = 2.705), in Logical Memory I–insignificant (*p* = 0.836, t = 0.208).

##### Visual Memory

In the visual memory domain, measured with Figure Recall from RBANS, the hypothesized group-time interaction was not observed (EG1 *p* = 0.619, t = 0.499, EG2 *p* = 0.643, t = 0.466).

##### Visuospatial/Constructional Function

In the last cognitive domain: visuospatial/constructional function, assessed with Line Orientation and Figure Copy from RBANS, the expected over-time improvement was observed only in one subtest in EG2: Figure Copy (EG1 *p* = 0.147, t = 1.466, EG2 *p* = 0.000, t = 3.639).

#### Secondary Outcome

##### Depressive Symptoms

The results show evidence of a decrease in depressive symptoms in the time-group comparison; however, the difference was statistically significant only in EG2 (EG1 *p* = 0.387, t = 0.871, EG2 *p* = 0.033, t = 2.165). Furthermore, taking into account the clinical interpretation of the scores obtained by subsequent participants, some participants in EGs changed their category from depression to norm (EG1 = 2 people, EG2 = 4 people), while one opposite case was observed in CG. However, the obtained results did not show a significant difference between EG1 and EG2 measured over time (*p* = 0.184, t = 1.339). Change in depression symptoms assessed with the use of the GDS scale was not associated with an improvement in cognitive performance in any of the cognitive domains enhanced after the intervention (MoCA *p* = 0.395, TMT A *p* = 0.954, Digit Span *p* = 0.952, 0 *p* = 0.667, Phonemic Fluency *p* = 0.333, List Recall *p* = 0.437, Logic Memory I *p* = 0.205). Detailed scores are described in Tables 2, 3.

**Table 2:** Primary and secondary outcome measures obtained in baseline and follow-up.

**Tool**	**Group**	**T1**			**T2**		
		**Mean (SD)**	**Median**	***p***	**Mean (SD)**	**Median**	***p***
MoCA	CG	25.40 (2.71)	25.0	0.8856	25.08 (2.84)	25.0	0.0676
	EG1	25.03 (2.29)	25.0		26.28 (2.07)	26.0	
	EG2	25.10 (2.66)	25.0		26.77 (2.60)	27.0	
Phonemic fluency MoCA	CG	15.44 (5.401)	15.0	0.6533	25.08 (2.84)	25.0	0.0138^*^
	EG1	15.90 (4.693)	16.0		26.28 (2.07)	26.0	
	EG2	14.83 (4.691)	14.5		26.77 (2.60)	27.0	
SCWT	CG	2.643 (0.58)	2.622	0.5194	2.576 (0.45)	2.50	0.5835
	EG1	2.904 (1.095)	2.731		2.637 (0.79)	2.43	
	EG2	2.914 (0.996)	2.774		2.857 (1.01)	2.75	
Figure copy RBANS	CG	19.80 (0.707)	20.0	0.0006^*^	19.72 (0.678)	20.0	0.4255
	EG1	19.17 (1.071)	20.0		19.52 (0.785)	20.0	
	EG2	18.67 (1.322)	19.0		19.63 (0.928)	20.0	
Line orientation RBANS	CG	16.72 (2.092)	17.0	0.6011	16.44 (1.583)	16.0	0.4711
	EG1	15.97 (2.946)	16.0		16.34 (2.742)	16.0	
	EG2	15.87 (4.041)	17.0		16.37 (3.908)	17.0	
Semantic fluency RBANS	CG	18.96 (4.809)	19.0	0.374	24.14 (6.010)	24.0	0.0031^*^
	EG1	21.17 (5.372)	20.0		18.84 (4.888)	19.0	
	EG2	20.63 (5.898)	21.5		23.53 (6.279)	24.5	
Picture naming RBANS	CG	9.640 (0.810)	10	0.7093	9.800 (0.645)	10	0.7838
	EG1	9.552 (8.783)	10		10.069 (1.791)	10	
	EG2	9.533 (0.900)	10		9.767 (0.504)	10	
List learning RBANS	CG	23.16 (7.587)	4.0	0.6535	24.80 (5.339)	5.0	0.0323^*^
	EG1	25.24 (5.813)	6.0		26.24 (5.276)	7.0	
	EG2	25.27 (6.175)	5.5		28.83 (5.954)	7.0	
List recall RBANS	CG	3.720 (2.807)	4.0	0.0499^*^	4.360 (2.612)	5.0	0.0065^*^
	EG1	5.586 (2.653)	6.0		6.034 (2.692)	7.0	
	EG2	4.733 (3.051)	5.5		6.733 (2.982)	7.0	
Logical memory I RBANS	CG	17.28 (3.553)	18.0	0.6852	16.32 (2.577)	18.00	0.0006^*^
	EG1	18.21 (3.266)	18.0		19.14 (2.560)	21.00	
	EG2	17.83 (3.940)	18.05		18.90 (3.252)	21.00	
Logical memory II RBANS	CG	14.83 (3.671)	15.0	0.9603	15.56 (2.755)	15.0	0.0006^*^
	EG1	13.07 (5.529)	14.0		16.10 (5.101)	18.0	
	EG2	13.37 (6.563)	15.0		15.20 (5.780)	17.5	
Figure recall RBANS	CG	14.48 (3.671)	15.0	0.6032	15.56 (2.755)	18.0	0.2642
	EG1	13.07 (5.529)	14.0		16.10 (5.101)	20.0	
	EG2	13.37 (6.563)	15.0		15.20 (5.780)	20.0	
TMT part A	CG	62.48 (21.71)	59.0	0.1146	60.48 (19.93)	59	0.0042^*^
	EG1	56.07 (28.34)	43.0		47.90 (22.12)	43	
	EG2	53.43 (20.14)	44.0		46.43 (14.80)	44	
GDS	CG	8.40 (5.737)	12.0	0.8297	9.480 (6.063)	12.0	0.0374^*^
	EG1	8.759 (5.520)	12.0		8.793 (5.809)	12.0	
	EG2	7.733 (5.112)	11.5		6.233 (4.352)	9.0	
		**Score**	**Percent**	**p**	**Score**	**Percent**	**p**
Digit span MoCA	CG	0	0%	1	0	8.00%	0.3927
		1	40.00%		1	36.00%	
		2	60.00%		2	56.00%	
	EG1	0	0%		0	3.45%	
		1	39.29%		1	24.14%	
		2	60.71%		2	72.41%	
	EG2	0	3.33%		0	3.33%	
		1	40.00%		1	16.67%	
		2	56.67%		2	80.00%	
Serial sevens MoCA	CG	0	4.00%	0.794	0	0%	0.3084
		1	20.00%		1	24.00%	
		2	76.00%		2	76.00%	
	EG1	0	6.90%		0	0%	
		1	24.14%		1	10.35%	
		2	68.97%		2	89.65%	
	EG2	0	3.33%		0	0%	
		1	33.33%		1	10%	
		2	63.33%		2	90%	

*Values are given as means (SD) or n (%). EG1, experimental group with Computerized Cognitive Training; EG2, experimental group with Computerized Cognitive Training and Whole Body Cryotherapy; CG, control group; MoCA, Montreal Assessment Cognitive Scale; GDS, Geriatric Depression Scale; SCWT, The Stroop Color and Word Test; RBANS, Repeatable Battery for the Assessment of Neuropsychological Status; TMT A, Trail Making Test part A.*

* *Asterisk indicates statistically significant score (p ≤ 0.05)*.

**Table 3:** Linear mixed model analysis results (T1, T2).

**Tool**	**Interaction description**	**Linear mixed model analysis–interaction effect**				
		**Estimate**	**Std. Error**	**Df**	**T Value**	**Pr(>|T|)**
Moca	(Intercept)	25.400	0.506	104.7	50.204	0.000^*^
	T2	−0.320	0.365	81.0	−0.877	0.383
	EG1	−0.366	0.690	104.7	−0.529	0.598
	EG2	−0.300	0.685	104.7	−0.438	0.662
	T2 × EG1	1.561	0.498	81.0	3.137	0.002^*^
	T2 × EG2	1.987	0.494	81.0	4.023	0.000^*^
	T2 EG1 × EG2	−0.425	0.475	81.0	−0.895	0.373
Phonemic fluency	(Intercept)	15.440	1.013	108.6	15.242	0.000^*^
	T2	−0.960	0.783	81.0	−1.225	0.224
	EG1	0.457	1.382	108.6	0.330	0.742
	EG2	−0.607	1.372	108.6	−0.442	0.659
	T2 × EG1	3.891	1.069	81.0	3.640	0.000^*^
	T2 × EG2	3.227	1.061	81.0	3.042	0.003^*^
	T2 EG1 × EG2	0.664	1.020	81.0	0.651	0.517
SCWT	(Intercept)	2.676	0.166	87.58	16.082	0.000^*^
	T2	−0.133	0.066	83.00	−2.023	0.046^*^
	EG1	0.161	0.223	81.00	0.725	0.471
	EG2	0.276	0.221	81.00	1.252	0.214
	T2 EG1 × EG2	−0.210	0.157	81.0	−1.340	0.184
Figury copy RBANS	Intercept)	19.800	0.191	141.3	103.558	0.000^*^
	T2	−0.080	0.212	81.0	−0.377	0.707
	EG1	−0.628	0.261	141.3	−2.405	0.017^*^
	EG2	−1.133	0.259	141.3	−4.378	0.000^*^
	T2 × EG1	0.425	0.290	81.0	1.466	0.147
	T2 × EG2	1.047	0.288	81.0	3.639	0.000^*^
	T2 EG1 × EG2	−0.622	0.277	81.0	−2.248	0.027^*^
Line orientation RBANS	(Intercept)	16.720	0.616	99.43	27.157	0.000^*^
	T2	−0.280	0.396	81.00	−0.707	0.481
	EG1	−0.754	0.840	99.43	−0.898	0.371
	EG2	−0.853	0.834	99.43	−1.024	0.308
	T2 × EG1	0.659	0.540	81.00	1.220	0.226
	T2 × EG2	0.780	0.536	81.00	1.455	0.149
	T2 EG1 × EG2	−0.121	0.515	81.00	−0.234	0.815
Semantic fluency RBANS	(Intercept)	18.960	1.122	107.5	16.895	0.000^*^
	T2	−0.120	0.852	81.0	−0.141	0.888
	EG1	2.212	1.531	107.5	1.445	0.151
	EG2	1.673	1.519	107.5	1.101	0.273
	T2 × EG1	3.086	1.162	81.0	2.655	0.010^*^
	T2 × EG2	3.020	1.153	81.0	2.619	0.011^*^
	T2 EG1 × EG2	0.066	1.109	81.0	0.059	0.953
Picture naming RBANS	(Intercept)	9.640	0.201	149.3	47.960	0.000^*^
	T2	0.160	0.239	81.0	0.669	0.506
	EG1	−0.088	0.274	149.3	−0.322	0.748
	EG2	−0.107	0.272	149.3	−0.392	0.696
	T2 × EG1	0.357	0.327	81.0	1.094	0.277
	T2 × EG2	0.073	0.324	81.0	0.226	0.822
	T2 EG1 × EG2	0.284	0.312	81.0	0.911	0.365
List learning RBANS	(Intercept)	23.160	1.210	104.8	19.146	0.000^*^
	T2	1.640	0.874	81.0	1.876	0.064
	EG1	2.081	1.651	104.8	1.261	0.210
	EG2	2.107	1.638	104.8	1.286	0.201
	T2 × EG1	−0.640	1.193	81.0	−0.537	0.593
	T2 × EG2	1.927	1.184	81.0	1.628	0.107
	T2 EG1 × EG2	−2.567	1.138	81.0	−2.255	0.027^*^
List recall RBANS	(Intercept)	3.720	0.562	109.4	6.616	0.000^*^
	T2	0.640	0.441	81.0	1.453	0.150
	EG1	1.866	0.767	109.4	2.432	0.017^*^
	EG2	1.013	0.761	109.4	1.331	0.186
	T2 × EG1	−0.192	0.601	81.0	−0.319	0.751
	T2 × EG2	1.360	0.596	81.0	2.280	0.025^*^
	T2 EG1 × EG2	−1.552	0.574	81.0	−2.705	0.008^*^
Logical memory I RBANS	(Intercept)	17.280	0.648	108.5	26.659	0.000^*^
	T2	−0.960	0.500	81.0	−1.919	0.058
	EG1	0.927	0.884	108.5	1.048	0.297
	EG2	0.553	0.878	108.5	0.630	0.530
	T2 × EG1	1.891	0.683	81.0	2.770	0.007^*^
	T2 × EG2	2.027	0.677	81.0	2.992	0.004^*^
	T2 EG1 × EG2	−0.136	0.651	81.0	−0.208	0.836
Logical memory II RBANS	(Intercept)	7.577	0.620	87.45	12.223	0.000^*^
	T2	0.845	0.243	83.00	3.481	0.001^*^
	EG1	0.414	0.830	81.00	0.499	0.619
	EG2	0.383	0.823	81.00	0.466	0.643
	T2 EG1 × EG2	−0.063	0.575	81	−0.110	0.913
Figure recall RBANS	(Intercept)	14.840	1.031	98.21	14.389	0.000^*^
	T2	0.720	0.643	81.00	1.120	0.266
	EG1	−1.771	1.407	98.21	−1.258	0.211
	EG2	−1.473	1.396	98.21	−1.055	0.294
	T2 × EG1	2.314	0.877	81.00	2.639	0.010^*^
	T2 × EG2	1.113	0.870	81.00	1.280	0.204
	T2 EG1 × EG2	1.201	0.837	81.00	1.436	0.155
TMT part A	(Intercept)	64.438	4.073	89.52	15.819	0.000^*^
	T2	−5.917	1.823	83.00	−3.246	0.002^*^
	EG1	−9.497	5.417	81.00	−1.753	0.083
	EG2	−11.547	5.376	81.00	−2.148	0.035^*^
	T2 EG1 × EG2	−1.172	4.350	81.0	−0.270	0.788
		**Estimate**	**Std. Error**	**z**	**Odds ratio**	**Pr(>|T|)**
Digit span from MoCA	0|1	−4.760	1.006	−4.734	0.009	0.000^*^
	1|2	−0.791	0.630	−1.255	0.453	0.210
	T2	−0.550	0.691	−0.796	0.577	0.426
	EG1	0.027	0.844	0.032	1.027	0.975
	EG2	−0.315	0.826	−0.381	0.730	0.703
	T2 × EG1	1.171	0.979	1.196	3.227	0.232
	T2 × EG2	2.085	1.018	2.049	8.041	0.041^*^
	EG1 × EG2	−0.913	0.976	−0.935	0.401	0.350
Serial sevens MoCA	1|2	−4.554	1.225	−3.716	0.011	0.000^*^
	2|3	−1.193	0.718	−1.662	0.303	0.097
	czasT2	1.560	0.579	2.697	4.761	0.007^*^
	EG1	0.226	0.783	0.288	1.253	0.773
	EG2	−0.006	0.771	−0.008	0.994	0.993
	EG1 × EG2	0.058	1.253	0.046	1.261	0.963

*Values are given as means (SD). EG1, experimental group with Computerized Cognitive Training; EG2, experimental group with Computerized Cognitive Training and Whole Body Cryotherapy; CG, control group; MoCA, Montreal Assessment Cognitive Scale; GDS, Geriatric Depression Scale; SCWT, The Stroop Color and Word Test; RBANS, Repeatable Battery for the Assessment of Neuropsychological Status; TMT A, Trail Making Test part A.*

* *Asterisk indicates statistically significant score (p ≤ 0.05)*.

## Discussion

In the past decade, we have been observing increasing interest in developing non-pharmacological procognitive interventions aimed at maintaining cognitive performance and delaying the onset of dementia. In the present study, we evaluated a combination of two different stimulating interventions: CCT and WBC administered to individuals with MCI and cognitive norm. The results indicated that CCT, especially combined with WBC, may lead to the improvement of already deteriorated cognitive functions. Moreover, combining CCT with WBC seems to result in a reduction of depressive symptoms.

In the case of global cognitive functioning measured by MoCA, we observed significantly higher scores in T2 in both EGs in relation to CG. In the assessment of specific cognitive domains, we also observed some positive changes. In EG1, the group in which only CCT was conducted, the improvement was particularly visible with regard to: attentional control (the ability to concentrate), sustained attention (the ability to maintain concentration) (Digit Span from MoCA), psychomotor speed (TMT: part A), verbal productivity (Sematic Fluency from RBANS and Phonemic Fluency from MoCA) and immediate memory (Logical Memory I from RBANS). The present findings are thus compliant with the nature of the Mind Academy® CCT, which focuses on such cognitive functions as attention (selective, sustained, divided, and executive), various memory and planning tasks and psychomotor speed. What is more, the group format of the training and the fact that the participants were given home tasks, which were presented publicly afterwards, resulted in increased social interaction, which might have led to the observed improvement in verbal fluency. The results are also consistent with some other studies on the effectiveness of CCT, which reported improvement in global functioning (40, 41), processing speed (42), immediate memory (41, 43, 44), and phonological fluency (45).

Moreover, the results of our study suggest that combining CCT with WBC leads to a slight enhancement of the procognitive effect of CCT as in two subtests (Figure Copy and List Recall from RBANS) the difference in the performance in T2 was statistically significant, in favor of EG2 (CCT + WBC). What is more, the impact of CCT alone on depressive symptoms was very limited, and the combination of CCT and WBC allowed us to address this issue, since a reduction in depressive symptoms was observed in the combined intervention, but not in CCT alone. This indicates that WBC affects the mood regardless of social interactions. Our results are in line with the results of other studies aimed at addressing alterations of mood after cryotherapy sessions (19, 46, 47). Seeing that the change in depression symptoms assessed by the GDS scale was not associated with an improvement in cognitive performance in any of the cognitive domains enhanced after the intervention, we may conclude that the improvement was probably caused by the intervention itself, not by the reduction of depressive symptoms.

Notably, the results of this study could suggest that WBC may improve cognition in humans. However, the exact mechanisms underlying the impact of cryogenic temperature on cognitive functioning requires further investigation (29). As current research suggests (48, 49), BDNF (brain-derived neurotrophic factor) could be a potential mediator of this effect. BDNF is a growth factor of the central nervous system, crucial for neuronal development and maintaining proper brain functions, such as cognition (50). By activating the pathways responsible for the regulation of neuron survival, BDNF acts in a neuroprotective way and helps prevent or inhibit the pathophysiological processes leading to cognitive deterioration (48, 49). Therefore, we recommend further research on the effectiveness of WBC, in which BDNF will be measured before and after the WBC/WBC + CCT intervention.

There were several limitations to the current study, apart from the relatively small sample and the lack of randomized allocation to the experimental groups and the control group. Firstly, it was not designed to examine the stability of CCT and WBC benefits over time. Secondly, it was a single-blinded trial; while it seems impossible to blind the trainee and participants, given the nature of the interventions, it nevertheless introduces the risk of expectation bias. Thirdly, it was difficult to distinguish specific contributions of the psychoeducation part and group tasks to cognitive improvement in participants. The Mind Academy® was intentionally designed as a multifaceted, enjoyable cognitive training that acquaints participants with knowledge of health-aging rules, and specific mnemotechnics. Therefore, we cannot conclude whether CCT used individually and without the psychoeducational part is as effective as CCT used in a group setting in combination with psychoeducation. Fourthly, we should note that all participants of the project were seniors purposely seeking stimulating activities. Keeping in mind that volunteers for research projects tend to be highly educated with higher socio-economic status (51), less anxious (52), and even healthier (53, 54) than general population, we should be aware of the risk of volunteer bias in the study sample. Fiftly, we choose 14 different subtest (two for each cognitive domain) for cognitive function assessment and the abundance of them may have increased a risk of making a type I error (“false positivity”). On the other hand, given concerns about statistical power (related to relatively low sample size in our study), we opted not to use correction for multiple tests in order to avoid type II error (“false negativity”). At the same time, the most prominent asset of the study was the fact that it assessed the effectiveness of two innovative approaches to cognitive function enhancement. Both approaches possess documented distinct mechanisms of impacting human brain. CCT is believed to induce the formation of new neuronal connections (neuroplasticity) and maintain the already existing pathways, while, WBC targets the neurodegenerative process by means of regulating the production of inflammatory mediators, such as cytokines IL-6 and IL-10 (55), nitric oxide (NO) (22). These mediators are released as a consequence of amyloid-beta deposition and the inflammatory response caused by the deposits. To our knowledge, no intervention study combining these two procognitive approaches and investigating whether WBC may enhance CCT's effectivity has been conducted before.

Moreover, unlike most related studies that focus on people already having major cognitive problems, we included only high-risk individuals. Accordingly, we could evaluate the effectiveness of CCT/CCT + WBC in seniors who experience slight cognitive decline and seek beneficial methods of slowing down the deteriorative process. What is more, the methods proposed by us are safe, widely available and low-cost, enabling their common use and implementation as a non-pharmacological intervention aimed at maintaining good cognitive performance.

In this study, we examined the effectiveness of Computerized Cognitive Training (CCT) combined with Whole Body Cryotherapy (WCB) in improving cognitive functions in older adults. We compared the results of patients from three groups: two experimental groups–EG1, in which CCT was used alone, EG2, in which interventions (CCT + WBC) were combined, and a control group, which included patients receiving usual care. After examining the results, the statistical analysis conducted by us showed that after the intervention the performance in the assessment of general cognitive functioning increased in both EGs. A significant improvement was also visible in several cognitive domains such as: verbal productivity, learning ability, and immediate memory, delayed memory, attentional control and information processing at T2 compared to T1 (*p* ≤ 0.05). Nevertheless, only in the group with combined interventions (CCT + WBC) the participants presented significantly less depressive symptoms (*p* ≤ 0.05), and we claim that in order to address cognitive decline and mood decline, CCT, and WBC ought to be combined.

Future studies should shed more light on the sustainability of benefits obtained as a result of procognitive interventions (longitudinal assessment). Additionally, they should investigate whether the transfer of skills acquired during the training occurs e.g., depending on whether the participants who have undergone CCT/CCT + WBC are more cognitively efficient in their every-day life. Further, well-designed randomized clinical trials on WBC and WBC + CCT would help provide more definitive evidence regarding the efficacy of these methods. Additionally, more investigation (e.g., in animal models) is needed in order to determine the mechanisms underlying WBC, namely the pathway linking exposure to extreme cold to the regulation of the inflammatory response.

## Data Availability Statement

The original contributions presented in the study are included in the article/supplementary material, further inquiries can be directed to the corresponding author/s.

## Ethics Statement

The studies involving human participants were reviewed and approved by Bioethical Committee (number KB61/218) at Wroclaw Medical University. The patients/participants provided their written informed consent to participate in this study.

## Author Contributions

AS and RW conceived and planned the study. AS, DS, and RW carried out the study. AS took the lead in writing the manuscript. All authors contributed to the interpretation of the results, provided critical feedback, and helped to shape the research, analysis, and manuscript.

## Conflict of Interest

The authors declare that the research was conducted in the absence of any commercial or financial relationships that could be construed as a potential conflict of interest.

## Abbreviations

WBC: whole-body cryotherapy
CCT: computerized cognitive training
EG: experimental group
CG: control group
SCD: subjective cognitive decline
MoCA: Montreal Assessment Scale
TMT: Trail Making Test
RBANS: Repeatable Battery for the Assessment of Neuropsychological Status
SCWT: The Stroop Color and Word Test
GDS: Geriatric Depression Scale
BDNF: brain-derived neurotrophic factor
NO: nitric oxide.
